# Spatial patterns and the associated factors for breast cancer hospitalization in the rural population of Fujian Province, China

**DOI:** 10.1186/s12905-023-02336-w

**Published:** 2023-05-09

**Authors:** Mengjie Song, Xiaoxi Huang, Xueqiong Wei, Xuwei Tang, Zhixiang Rao, Zhijian Hu, Haomin Yang

**Affiliations:** 1 Department of Epidemiology and Health Statistics, School of Public Health & Key Laboratory of Ministry of Education for Gastrointestinal Cancer, Fujian Medical University, Fuzhou, 350122 China; 2 Department of Breast, Fujian Maternity and Child Health Hospital, College of Clinical Medicine for Obstetrics & Gynecology and Pediatrics, Fujjan Medical University, Fuzhou, 350001 China; 3 School of Geographical Sciences, Nanjing University of Information Science and Technology, Nanjing, 210044 China; 4 Department of Medical Epidemiology and Biostatistics, Karolinska Institute, Stockholm, 17177 Sweden; 5 Department of Epidemiology and Health Statistics, School of Public Health, Fujian Medical University, University Town, Xue Yuan Road 1, Fuzhou, 350122 China

**Keywords:** Soil heavy metals, Socioeconomic factors, Breast cancer hospitalization, Spatial heterogeneity, GWPR

## Abstract

**Background:**

Despite the known increasing incidence of breast cancer in China, evidence on the spatial pattern of hospitalization for breast cancer is scarce. This study aimed to describe the disparity of breast cancer hospitalization in the rural population of Southeast China and to explore the impacts of socioeconomic factors and heavy metal pollution in soil.

**Methods:**

This study was conducted using the New Rural Cooperative Medical Scheme (NRCMS) claims data covering 20.9 million rural residents from 73 counties in Southeast China during 2015–2016. The associations between breast cancer hospitalization and socioeconomic factors and soil heavy metal pollutants were evaluated with quasi-Poisson regression models and geographically weighted Poisson regressions (GWPR).

**Results:**

The annual hospitalization rate for breast cancer was 101.40/100,000 in the studied area and the rate varied across different counties. Overall, hospitalization for breast cancer was associated with road density (β = 0.43, *P* = 0.02), urbanization (β = 0.02, *P* = 0.002) and soil cadmium (Cd) pollution (β = 0.01, *P* = 0.02). In the GWPR model, a stronger spatial association of Cd, road density and breast cancer hospitalization was found in the northeast regions of the study area while breast cancer hospitalization was mainly related to urbanization in the western regions.

**Conclusions:**

Soil Cd pollution, road density, and urbanization were associated with breast cancer hospitalization in different regions. Findings in this study might provide valuable information for healthcare policies and intervention strategies for breast cancer.

**Supplementary Information:**

The online version contains supplementary material available at 10.1186/s12905-023-02336-w.

## Introduction

Female breast cancer is a leading cause of cancer in the world and in China [1]. Nowadays, the increasing health burden of breast cancer has become a critical public health issue in China, with cases accounting for 9.1% of all newly diagnosed cancers and 3.9% of all cancer deaths in China [2]. More importantly, the distribution of breast cancer in China varies greatly [3], contributing to a difference in hospitalization for breast cancer [4, 5]. While such geographic disparities might be influenced by the differences in the distribution of environmental exposures and socioeconomic factors [6–8], previous evidence was, however, usually limited by the assumption of constant estimates over the entire geographic area and unable to capture the geographical variations in the relationships [9, 10].

Socioeconomic factors could influence women’s lifestyle [11], including dietary pattern, physical activity and reproductive behavior [12], which are known factors for breast cancer risk [1, 6, 13, 14] and hospitalization [15]. In addition, recent evidence suggests that hospitalizations for breast cancer might also be influenced by travel distances [16]and social security [17]. However, no study has systemically assessed the potential role of indicators for socioeconomic development and breast cancer hospitalization.

Moreover, special attention should be paid to the impact of environmental exposures, especially soil heavy metal pollution, considering the biological [18, 19] carcinogenesis effects from heavy metals. As heavy metals in soil are difficult to break down [20], soil heavy metals could serve as an indicator of long-term exposure to heavy metals [21]. While previous studies mainly focused on the associations between dietary heavy metals and breast cancer [22–26], there is less information on the association between chronic exposure to heavy metals in soil and breast cancer hospitalization, especially in the rural population who mainly face to the soil heavy metals.

Assessment of the spatial pattern and associated factors for breast cancer hospitalization is important for the planning of cancer-related health resources and identifying priority regions for possible prophylactic interventions. In this study, we focus on the spatial autocorrelation of breast cancer hospitalization in the rural population of Southeast China and explore the association between socioeconomic factors, soil heavy metals and breast cancer hospitalization by using general spatial autocorrelation. We further used the geographically weighted Poisson regression (GWPR) model to explore spatial variations of the associations between socioeconomic factors, soil heavy metals and breast cancer.

## Materials and methods

### Data sources

This study used data from the New Rural Cooperative Medical Scheme (NRCMS) in Fujian Province, which is located in the southeast of China (ranging from 23°33′ ~ 28°20′ N to 115°50′~ 120°40′ E). It covers an area of about 124,000 km ^2^, with a total population of 37.2 million in 2015, of which 20.9 million were rural. By the end of 2014, 98.9% of the rural population (https://data.cnki.net/yearBook/single?id=N2015110123) participated in the NRCMS. Therefore, we extracted medical insurance claims data of hospitalized female breast cancer patients within the NRCMS from January 1, 2015, to December 31, 2016. Due to the small number of rural residents in Taijiang County, Gulou County, Licheng County, Fengze County, Cangshan County, and Xiamen City, people in these counties were covered by the Urban Basic Medical Insurance and were excluded from the analysis, leaving 73 counties covering about 98% of the rural population in the study area.

Breast cancer hospitalization was identified using the International Classification of Diseases (ICD) 10 code C50. The hospitalization data consisted of age, sex, county of registered residence, year of hospitalization, and cause of hospitalization. Cases were selected based on the county of registered residence rather than hospital attended, which ensures a better representation of the spatial distribution of the exposed population to the environmental and socioeconomic factors. The rural female population data was obtained from the Fujian Statistical Yearbook (https://data.cnki.net/yearBook/single?id=N2017020245). All data were aggregated at the county level. The rate of breast cancer hospitalization was then calculated as the number of female breast cancer hospitalizations divided by female rural population in 73 counties.

### Socioeconomic factors

Socioeconomic factors included population density, urbanization level, road density, per capita GDP, medical institutions, and health workers at the county level. The population density was defined as the number of people per square kilometer in each county. Urbanization level was defined as the proportion of people who live in urban areas among the entire population in each county. Road density was defined as the length of road per square kilometer in each county. Per capita GDP was defined as the ratio of the gross domestic product realized to the resident population in each county. Medical institutions were defined as the number of medical institutions per 1000 population in each county. Health workers were defined as the number of health workers per 1000 population in each county. We used the average level of these covariates during 2015–2016 to estimate their association with breast cancer hospitalization (supplementary Data 1 and Supplementary Table 1).

### Soil heavy metals

In this study, the data on soil heavy metals was obtained from the Central Station of Environmental Monitoring of Fujian Province. The details of the sample collection and testing can be found in Chen’s study [27]. Briefly, all samples were from the forest land, grassland, orchard, tea garden, paddy field, dry land and tidal flat land in the map of land-use types in Fujian Province, and the grid was divided based on the geographic information system platform. The tea, orchards and arable land were laid out with a grid density of 8 km×8 km. For arable land in some key agricultural areas, 4 km×4 km of densely laid points were used. For tidal flats, one point was laid every 16 km along the coastline. Specifically, a total of 1114 surface soil samples were collected from the study area. The spatial distributions of heavy metals were estimated in terms of the ordinary kriging technique based on point data. ArcMap software was used for image registration and calculating pollutant regions of soil heavy metals at the county level. Supplementary Table 2 presents the descriptive statistics of soil heavy metals.

### Statistical analysis

#### Spatial autocorrelation analysis

To explore the spatial distribution pattern of hospitalizations for breast cancer, the global Moran’s I index and the local Moran index were used in this study [28]. The global Moran’s I index evaluated the spatial autocorrelation degree of the entire study area, while the local Moran statistics result, namely, local indicators of spatial association (LISA), was used to explore the existence of anomalies in local areas [29]. The corresponding formulas [30] are as follows:

$$I = \frac{{\Sigma \begin{array}{*{20}{c}}k\\{i = 1}\end{array}\Sigma \begin{array}{*{20}{c}}k\\{j = 1}\end{array}({X_i} - \bar x)({X_j} - \bar x)}}{{{S^2}\Sigma \begin{array}{*{20}{c}}k\\{i = 1}\end{array}\Sigma \begin{array}{*{20}{c}}k\\{j = 1}\end{array}{W_{ij}}}}$$

1 $${I^*} = \Sigma \begin{array}{*{20}{c}}m\\{p \ne q}\end{array}{W_{pq}}{Z_p}{Z_q}$$

In the formulation, I represents the global Moran’s I index; k represents the number of grids. X_i_ and X_j_ stand for the values of grid i and grid j, and W_ij_ is the spatial weight matrix between grid i and grid j (W_ij_ = 1 if grid i is adjacent with grid j, and W_ij_ = 0 otherwise). S^2^ is the variance of the whole grid. I* is the local Moran’s I index; Wpq is the standardized spatial weight matrix; and Zp and Zq represent standardized values of grid p and q.

### Associations with socioeconomic factors and soil metal pollution

Quasi Poisson regression model was used to evaluate the overall association between the socioeconomic variables (Urbanization, Road density, Population density, Health workers, Medical institutions and Per capita GDP) and breast cancer hospitalization in women. In the univariate model, we tested the association with each of these variables, while the multivariable model mutually adjusted for the statistically significant variables identified in the univariate model.

Then, we selected statistically significant socioeconomic variables in the multivariable model, and incorporated each soli heavy metal pollutant into the model, separately [31]. This implementation allowed a global assessment of the existence of relationships, as well as their directions, between the different covariates and the rate of breast cancer hospitalization in women before analyzing the effect of the association by county.

In the next step, geographically weighted Poisson regression was used to identify the factors associated with breast cancer hospitalization. The geographical weight regression model allows the association between two factors to vary spatially, which has been used in several previous environmental studies [32–35]. The model of GWPR is shown in Eq. (2),

2 $$\begin{array}{l}{y_i} \sim Poisson\left[ {{N_i}exp\left( {{u_i}} \right)} \right]\\{u_i}(\{ {x_{k,i}}\} ) = {\Sigma _k}{\beta _k}({u_i},{v_i}){x_{k,i}}\end{array}$$

where y_i_, u_i_, x_k, i_ and N_i_ are, respectively, dependent variable (the number of women who were hospitalized for breast cancer), the linear predictor, kth independent variable including the constant term, and the offset variable corresponding to population size at risk (defined as the average rural female population in 2015 and 2016) at the location i. It should be noted that the estimated risk of hospitalization for breast cancer at location i is given by the term of exp(u_i_). (u_i_, v_i_) is the x-y coordinate of the ith location; and coefficients β_k_(u_i_, v_i_) are assumed to be smoothly varying conditional on the location.

The estimates of the local coefficients at the location i are obtained by fitting a usual Poisson regression model to the data subset around the regression point i with a geographical weighting function. The standard errors of estimated local coefficients can be derived using local regression theory. A descriptive measure of goodness-of-fit for Poisson regression is the percent of deviance explained:

$${\rm{ pde}}{\upsilon _i}{\rm{ = 1 - de}}{\upsilon _i}{\rm{/nullde}}{\upsilon _i}$$

The data were screened and cleaned using exploratory data analysis using SPSS26.0 and Microsoft Excel. Global Moran’s I, LISA values, were measured using ArcMap software (version 10.6). We used GWR software (version 4.0.80) to implement a GWPR to explore spatial non-stationarity in our data. All vector maps were created using ArcMap software (version 10.6). The study was approved by the ethical committee at Fujian Medical University (committee’s reference number 2019-27).

## Result

### Spatial distribution of breast cancer hospitalizations and socioeconomic factors

**Fig. 1:**
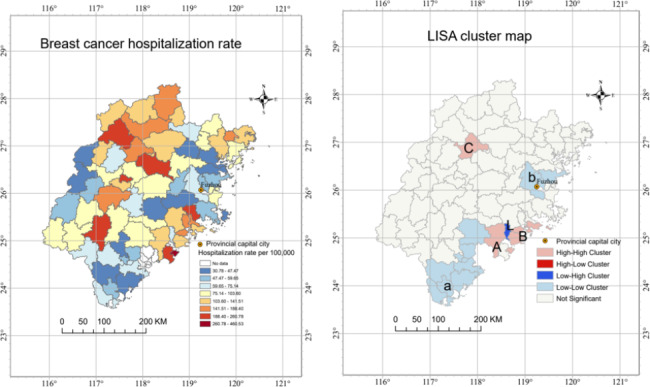
Breast cancer hospitalization rate and LISA cluster map of 73 counties in southeast China

Table 1 illustrates the demographic characteristics of hospitalized breast cancer patients. The mean rate of breast cancer hospitalization in the rural population of Fujian Province was 101.40/10^5^. The spatial distribution map (Fig. 1, left) shows that the values of breast cancer hospitalization rates in each county in the study area have obvious spatial aggregation.

**Table 1:** Demographic characteristics of breast cancer hospitalizations

Variable	No. of hospitalization	Proportion (%)
Total	20,375	100.00
Year		
2015	8466	41.55
2016	11,909	58.45
Age(year)		
< 30	450	2.21
30~	2600	12.75
40~	8185	40.12
50~	6393	31.34
> 60	2772	13.59
Hospital level		
Township	221	1.08
County	3643	17.88
Municipal	12,058	59.18
Provincial	4453	21.86
Length of stays (days)		
1–7	11,311	55.51
8–14	5387	26.44
≥ 15	3677	18.05

The Moran Index was calculated by using edges and corners as adjacent elements, and the global Moran index was 0.40 (p < 0.01), which suggested significant clustering. We further employed local Moran statistics to observe the local aggregation of breast cancer hospitalization rates. According to the result of local Moran statistics, two ‘high-high’ cluster regions, two ‘low-low’ cluster regions, as well as one ‘low-high’ cluster region were detected as statistically significant clusters (Fig. 1, right). From the LISA cluster map, the significant ‘high-high’ clusters A and B were located in southeastern Fujian. Additionally, another ‘high-high’ cluster C was located in central Fujian. The large clusters a and b with low risk existed in southern and mid-eastern Fujian, respectively. Moreover, there was ‘low-high’ cluster L between ‘high-high’ clusters A and B.

Table 2 displays the results of the Quasi-Poisson Regression model with socioeconomic factors. In univariate analyses, urbanization, population density and road density were significantly associated with breast cancer hospitalization rate. In the multivariable model, urbanization rate (β = 0.02, *P* = 0.002) and road density (β = 0.43, *P* = 0.02) were significantly associated with breast cancer hospitalizations. However, medical institutions, health workers and per capita GDP were not significantly associated with breast cancer hospitalization rate at the 0.05 level.

**Table 2:** Summary of parameters in the Quasi-Poisson regression model in socioeconomic variables

		Univariate								Multivariate				
Variable		Coefficient		Standard Error		p-value				Coefficient		Standard Error		p-value
Urbanization	0.023654*		0.004516		< 0.000001				0.017450*		0.005540		0.002440	
Medical institutions	0.000717		0.000393		0.072400				-		-		-	
Population density	0.000379*		0.000111		0.001020				-0.000271		0.000247		0.275010	
Road density	0.350000*		0.079940		0.000040				0.431900*		0.187600		0.024390	
Health workers	0.000696		0.000368		0.062800				-		-		-	
Per capita GDP	0.000001*		0.000003		0.001330				0.000001		0.000004		0.727190	

*Results statistically significant

### Spatial relationship between soil heavy metals and breast cancer hospitalization

**Fig. 2:**
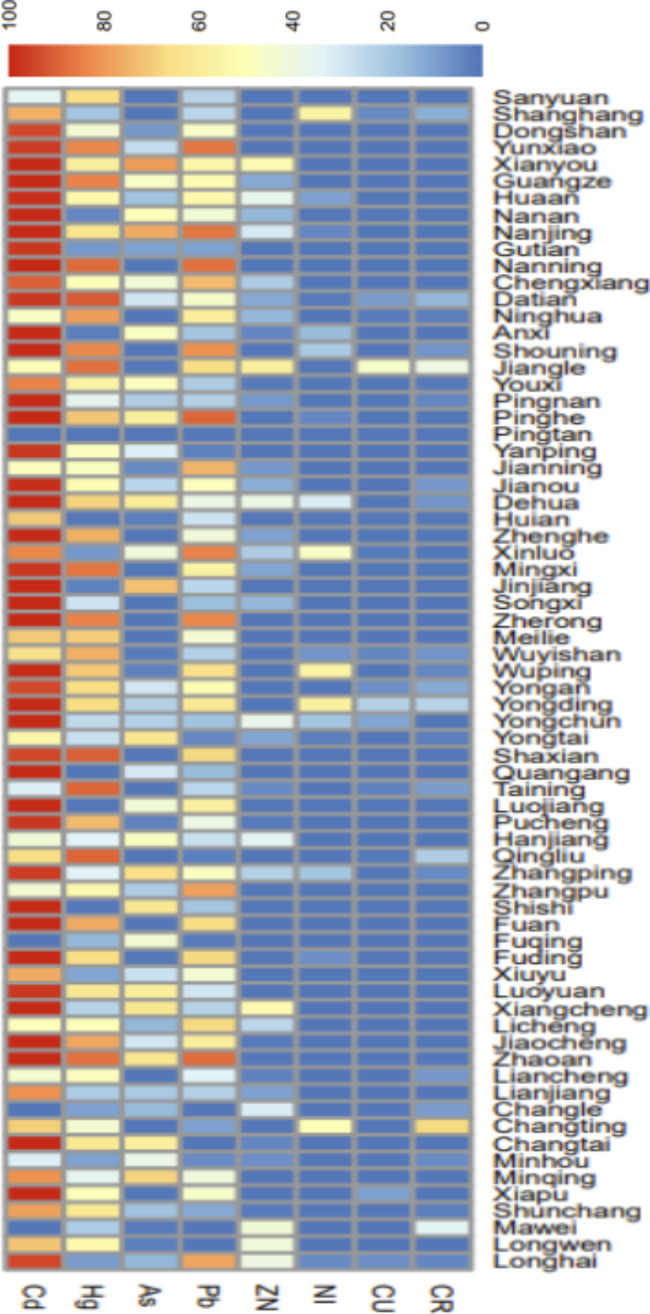
Percentage of the contaminated area of soil heavy metals in Fujian Province

In this study, ArcMap software was used to extract the polluted areas of arsenic (As), cadmium (Cd), chromium (Cr), copper (Cu), mercury (Hg), nickel (Ni), lead (Pb), and zinc(Zn) in these 73 counties. Cd was the most serious pollutant covering 54.27% of the total area, followed by Hg, As, and Pb. To eliminate the influence of the area factor of each county, the polluted area of each county was converted into the percentage of soil polluted area of each county. Distribution of percentage of polluted areas in 73 counties of Fujian province was visualized by color in Fig. 2.

**Fig. 3:**
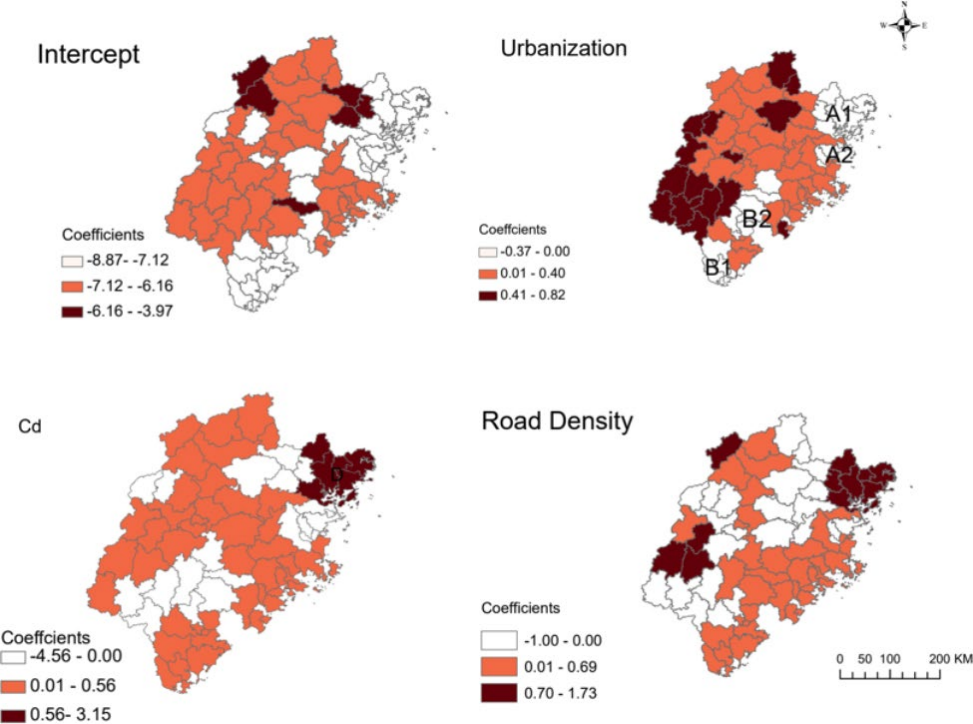
Parameters of predicting variables by county in the GWPR The red color on the map indicates a higher value of a local parameter estimate

After adjustment for urbanization and road density, Cd was the only heavy metal significantly associated with the hospitalization rate of breast cancer (β = 0.005, *P* = 0.016; Table 3). Using the GWPR analysis, the spatial variation of the estimated effects from urbanization, road density and Cd over the 73 counties is shown in Fig. 3. The association between breast cancer hospitalization and urbanization was the highest in the north and the west of Fujian Province, while the association with Cd was the highest in the northeast part. The strongest association between the hospitalization rate of breast cancer and road density was found in counties in Northeast and Southwest Fujian.

**Table 3:** Summary of parameters in the Quasi-Poisson regression model in soil heavy metals

Variable	Coefficient	Standard Error	p-value
As	0.001	0.002	0.781
Cd*	0.005	0.002	0.016
Cr	0.001	0.005	0.773
Cu	-0.004	0.011	0.740
Hg	0.000	0.000	0.103
Ni	-0.001	0.004	0.797
Pb	0.000	0.002	0.951
Hg	0.000	0.000	0.103

*Results statistically significant

Model adjusted for urbanization and road density

## Discussion

### Key results

In this study, we investigated the association of breast cancer hospitalization rates with socioeconomic factors and the spatial distribution of soil heavy metals at the county level in the rural population of Fujian Province, China. Urbanization and road density were positively associated with the hospitalization rate of breast cancer. We further used geographically weighted Poisson regression analysis to explore the spatial relationship between Cd, road density, urbanization and the hospitalization rate of breast cancer. We found a stronger spatial association of Cd, road density and breast cancer in the northeast of Fujian, while the spatial relationship between urbanization and breast cancer was in western Fujian.

Due to the different spatial distributions of environmental and socioeconomic factors, the distribution of disease presents dramatic spatial heterogeneity in geographic regions. Our study found a distinct pattern of breast cancer hospitalization in Fujian Province, characterized by significantly higher clusters in the southeast of Fujian versus lower clusters in southern regions. The spatial autocorrelation analysis further showed three high clusters (A, B, C), two low clusters (a,b), and one high-low cluster(L) for the hospitalization of breast cancer. This spatial distribution may be partly because of the uneven distribution of medical resources for the rural population in Fujian province, with most provincial and high-quality hospitals located in relatively developed coastal cities. However, it’s worth noting that the developing area (C) had a high hospitalization rate, suggesting stronger demand for medical resources and unequal resource distribution in this area. Recently, due to the rapid economic development in China [36], even people in developing areas in Fujian province have increased their incomes and improved health awareness of cancer. In addition, women have more frequent exposure to environmental pollutants (such as heavy metals, noise and dioxins) [37], resulting in an increased incidence of breast cancer and demand for related health services. Therefore, sufficient attention should be paid to the areas in need and immediate action should be taken to improve the fairness of medical resources allocation in Fujian province.

In this study, we found a positive association between urbanization and breast cancer hospitalization in the majority of the counties, which was consistent with the previously reported association between urbanization and breast cancer risk [38–43]. In recent years, more resources have shifted in China towards the development of small towns, especially in southeastern coastal areas of China, implying that these areas are experiencing a rapid development period [44]. Women in these areas may have a higher living standard, increased health awareness, and were more likely to afford breast cancer treatments. In addition, the development of economy may also lead to more life pressures, higher body mass index(BMI) [45], and changes in women’s lifestyle and reproductive behaviors [12, 46], which are known risk factors for breast cancer and may be responsible for the observed associations.

Together with the increase of urbanization, road density is also increasing in China recently. Positive association between road density and breast cancer hospitalization was found in many counties, possibly due to several reasons. First, higher road density means that there may be more traffic pollution. Studies on traffic-related pollutants have suggested that NO_2_ might be associated with a higher risk of breast cancer [47, 48]. PAHs, mainly originating from traffic, were also associated with breast cancer [49]. What’s more, one recent study from a Danish nationwide cohort found that road traffic noise was related to an increased risk of breast cancer [50]. Second, a higher road density also means more convenient transportation to medical institutions [51], which would consequently increase the number of hospitalizations. The strongest association between the low hospitalization rate of breast cancer and low road density in Southwest Fujian further supported this possibility, considering the quite limited medical resources in western Fujian. However, the strong association in Northeast Fujian might be the result of environmental pollution.

We observed substantial geographic heterogeneity in the association between Cd and breast cancer hospitalization, with the strongest association observed among women in the northeast region (region D). Cd has been classified as a known carcinogen [52]. The increased hospitalization for breast cancer in regions with higher Cd pollution is biologically plausible as Cd is involved in the carcinogenesis of breast cancer by activating ER [53, 54], inducing cell proliferation, differentiation, and apoptosis [55, 56], and modulating gene expression. Epidemiological studies have also indicated that urinary Cd levels were higher in breast cancer patients than in normal controls [57, 58], while breast cancer tissue had four times higher Cd levels than surrounding healthy tissue [31].

Considering the geographical disparity of risk factors for breast cancer hospitalization, different measures should be taken to improve women’s health. In western areas, which are in the rapid development stage, urbanization is the main risk factor for breast cancer and women have more demand for high quality medical resources. Health education to increase awareness of socioeconomic and lifestyle risk factors might reduce their future risk of breast cancer. In addition, regular screening services could be provided to detect breast cancer earlier and avoid hospitalizations caused by severe diseases. What’s more, for those coastal east areas, road density and Cd are the main risk factors. It is therefore vital to strengthen environmental governance, such as limiting the discharge of industrial heavy metals, especially Cd, and promoting the use of electric vehicles.

The major strength of our study is the use of geographically weighted regression models, which are able to detect the spatially varied magnitude of the associations and provide insight into priority regions for health resource planning and allocation. Moreover, we had access to a variety of data on socioeconomic factors and soil heavy metals, allowing us to explore the potential influencing factors.

Despite these, our findings should be interpreted in the context of several limitations. First, the design of ecological studies limits the ability to prove causality. The observed associations may be influenced by many unobserved confounding factors, such as other cultural factors, policy factors, and other natural factors. Second, the research unit of this paper is the county level divided by administrative regions. However, the distribution of environmental factors may be related to the natural geographical environment, and the corresponding effect might be attenuated. Subsequent studies are, therefore, needed to confirm the relationship between Cd and breast cancer using regions divided by the natural environment.

## Conclusion

Soil Cd pollution, road density, and urbanization were associated with breast cancer hospitalization in southeast China. The findings in this paper will help to understand the regional impact of Cd, road density and urbanization on the hospitalization rate of breast cancer, and provide valuable reference for the prevention strategy for breast cancer in southeast China and similar regions.

## Electronic supplementary material

Below is the link to the electronic supplementary material.

Supplementary Table 1 source of the used dataset
Supplementary Table 2 contaminated area of soil metal elements (km^2^)
Supplementary Table 3 Summary of local parameters in the GWPR
Supplementary Data 1 Socioeconomic data used in the analysis

## Data Availability

The hospitalization data that support the findings of this study are applied from the New Rural Cooperative Medical Scheme (NRCMS) database, and is therefore not publicly available. Part of the population dataset analyzed in this study can be found at https://data.cnki.net/yearBook/single?id=N2017020245, and provided in supplementary data 1. Data is, however, available from the corresponding author upon reasonable request and with permission of the Fujian Medical Security Bureau.
