# Supplementary material for: Spatial patterns and the associated factors for breast cancer hospitalization in the rural population of Fujian Province, China
Source: BMC Womens Health. 2023 May 9;23:247. doi: 10.1186/s12905-023-02336-w (PMC10170828; doi:10.1186/s12905-023-02336-w)
Supplement: Supplementary file 1 — Supplementary Table 1 source of the used dataset Supplementary Table 2 contaminated area of soil metal elements (km2) Supplementary Table 3 Summary of local parameters in the GWPR Supplementary Data 1 Socioeconomic data used in the analysis [file 12905_2023_2336_MOESM1_ESM.docx]

Supplementary Table 1 source of the used dataset

| Data name | Year | Spatiotemporal resolution | Source |
| --- | --- | --- | --- |
| NRCMS | 2015,2016 | - | - |
| Rural female population* | 2015,2016 | - | https://data.cnki.net/yearBook/single?id=N2017020245;  https://data.cnki.net/yearBook/single?id=N2017110017 |
| Population density* | 2015,2016 | - |  |
| Urbanization level* | 2015,2016 | - |  |
| Road density* | 2015,2016 | - |  |
| Per capita GDP* | 2015,2016 | - |  |
| Medical institutions* | 2015,2016 | - |  |
| Health workers* | 2015,2016 | - |  |
| Soil heavy metals* | 2013 | 4km*4km | https://www.cnki.com.cn/Article/CJFDTotal-FJDL201604007.htm |

Supplementary Table 2 contaminated area of soil metal elements（km^2^）

| Soil heavy metals | MIN | MEDIAN | MAX | percentage（%） |
| --- | --- | --- | --- | --- |
| As | 0.00 | 251.03 | 919.98 | 17.36 |
| Cd | 0.00 | 1108.94 | 1154.00 | 54.27 |
| Cr | 0.00 | 0.00 | 777.24 | 4.01 |
| Cu | 0.00 | 0.00 | 519.36 | 1.30 |
| Hg | 0.00 | 615.60 | 1057.95 | 33.52 |
| Ni | 0.00 | 0.00 | 669.03 | 4.09 |
| Pb | 0.00 | 484.21 | 1028.42 | 27.75 |
| Zn | 0.00 | 20.70 | 678.33 | 7.65 |

Percentage: the area of soil metal elements in Fujian Province

Supplementary Table 3 Summary of local parameters in the GWPR

| Variable | MIN | MEAN | STD | MAX |
| --- | --- | --- | --- | --- |
| Intercept | -8.87 | -6.89 | 0.70 | -3.97 |
| Cd | -4.57 | 0.04 | 0.92 | 1.72 |
| Urbanization | -0.38 | 0.22 | 0.25 | 0.82 |
| Road density | -1.00 | 0.23 | 0.54 | 1.73 |

Bandwidth size:12.52

Corrected Aikake information criterion(AICc): 423.14

Residual deviance: 209.40, Degree of freedom: 22.68

Supplementary Data 1 Socioeconomic data used in the analysis

| county | per capita GDP | population density | road density | urbanization | health workers | medical institutions |
| --- | --- | --- | --- | --- | --- | --- |
| sanyuan | 63377.5 | 121.36049 | 0.71623302 | 85 | 576.30603 | 705.224 |
| shanghang | 70993 | 182.75621 | 0.729276 | 44.849998 | 541.53497 | 495.42499 |
| dongshan | 75777.5 | 870.96771 | 1.55328 | 56.099998 | 394.63501 | 397.798 |
| yunxiao | 40299 | 433.25406 | 0.72029901 | 47.549999 | 338.98401 | 356.37299 |
| xianyou | 38073 | 621.75446 | 1.42834 | 42.150002 | 367.84399 | 342.64801 |
| guangze | 60233.5 | 73.63839 | 0.47589299 | 45.349998 | 548.284 | 470.26001 |

| huaan | 68625.5 | 131.69014 | 1.0289 | 50.25 | 245.48199 | | | 334.147 |
| --- | --- | --- | --- | --- | --- | --- | --- | --- |
| nanan | 58968 | 806.97736 | 1.6444 | 56.299999 | 270.16501 | | | 341.54401 |
| nanjing | 66518.5 | 184.17432 | 1.02062 | 49.049999 | 329.793 | | | 274.75 |
| gutian | 47336.5 | 181.4472 | 0.659958 | 42.599998 | 451.86301 | | | 432.03699 |
| zhouning | 43158 | 202.149 | 0.84240699 | 48.349998 | 459.71399 | | | 490.293 |
| chengxiang | 70337.5 | 810.39606 | 1.36255 | 69.400002 | 782.01599 | | | 644.12598 |
| datian | 52941 | 177.81012 | 0.76286 | 48.049999 | 443.93301 | | | 409.84698 |
| ninghua | 40548.5 | 156.99751 | 0.61611998 | 41.700001 | 448.49899 | | | 416.47501 |
| anxi | 44291.5 | 390.03925 | 1.32745 | 43.650002 | 318.36801 | | | 338.67801 |
| shouning | 40276 | 185.82455 | 0.979635 | 45.950001 | 438.74799 | | | 399.16 |
| jiangle | 70142.5 | 82.820168 | 0.53161198 | 52.599998 | 523.94702 | | | 507.31201 |
| youxi | 55420.5 | 129.33957 | 0.73202401 | 42.450001 | 468.48901 | | | 406.48499 |
| pingnan | 48231.5 | 129.22559 | 0.59533602 | 42.200001 | 465.87 | | | 528.80402 |
| pinghe | 36368 | 266.16882 | 0.686131 | 43.400002 | 264.26801 | | | 370.064 |
| pingtan | 45958.5 | 1167.8763 | 1.66307 | 45.349998 | 435.53601 | | | 312.759 |
| yanping | 60949.5 | 190.00754 | 0.83007503 | 66.849998 | 734.96399 | | | 803.62903 |
| jianning | 68767 | 91.317017 | 0.60971498 | 42.299999 | 382.84201 | | | 308.35199 |
| jianou | 46128 | 131.07726 | 0.32238099 | 48.799999 | 488.70499 | | | 522.66498 |
| jianyang | 51848.5 | 105.14336 | 0.76114601 | 55.700001 | 683.58502 | | | 602.45203 |
| dehua | 66249 | 149.75359 | 1.0062701 | 73.849998 | 453.56 | | | 471.78699 |
| huian | 78885.5 | 1487.1323 | 2.34304 | 55.799999 | 346.30801 | | | 360.20099 |
| zhenghe | 32141.5 | 134.74771 | 0.80388802 | 43.950001 | 557.67999 | | | 469.05301 |
| xinluo | 93017.5 | 193.24374 | 0.80552399 | 70.849998 | 1071.8101 | | | 884.42401 |
| mingxi | 60458.5 | 68.468208 | 0.64461398 | 50.349998 | 503.418 | | | 477.96301 |
| jinan | 62375 | 589.00604 | 0.86243403 | 99.25 | 645.62799 | | | 359.216 |
| jinjiang | 80942 | 1731.1538 | 3.1308401 | 64.599998 | 286.422 | | | 222.259 |
| songxi | 37518 | 160.83414 | 0.782359 | 44.599998 | 576.23798 | | | 553.90302 |
| zherong | 54513 | 200.82721 | 1.20588 | 60.049999 | 521.62402 | | | 537.70203 |
| meilie | 131981 | 403.9548 | 1.09065 | 97.800003 | 1399.79 | | | 947.841 |
| wuyi | 62255 | 86.24688 | 0.58209002 | 55.849998 | 568.71899 | | | 471.66199 |
| wuping | 55897 | 149.10918 | 0.64215302 | 46.549999 | 556.97998 | | | 609.58099 |
| yongan | 93027 | 113.30604 | 0.587695 | 66.949997 | 770.62 | | | 719.83899 |
| yongding | 57174.5 | 227.67535 | 1.10342 | 44.799999 | 490.323 | | | 482.048 |
| yongchun | 69424.5 | 404.63214 | 1.84146 | 58.150002 | 404.21799 | | | 453.46301 |
| yongtai | 54611 | 170.12032 | 0.85899198 | 39.650002 | 438.095 | | | 354.95999 |
| shaxian | 86431 | 148.91606 | 0.68154299 | 62.400002 | 568.48297 | | | 517.53003 |
| quangang | 113664 | 1321.4053 | 1.6209199 | 49.049999 | 464.33401 | | | 388.92001 |
| taining | 77313 | 89.764549 | 0.618182 | 48.150002 | 665.078 | | | 561.901 |
| luojiang | 70671.5 | 494.09448 | 1.32723 | 55.75 | 331.25699 | | | 284.29099 |
| pucheng | 42667 | 128.67299 | 0.56488299 | 45.849998 | 474.97 | | | 563.52502 |
| hanjing | 85827 | 556.69586 | 1.5757999 | 78.400002 | 821.70398 | | | 876.29199 |
| qingliu | 62493.5 | 86.655594 | 0.481644 | 45.700001 | 516.48401 | | | 411.59399 |
| zhangping | 80976 | 99.529572 | 0.60887098 | 53.950001 | 535.38898 | | | 600.40802 |
| zhangpu | 40358.5 | 422.08762 | 0.82041001 | 49.450001 | 350.45999 | | | 326.88901 |
| shishi | 101089 | 1852.809 | 3.4437499 | 78.199997 | 418.15399 | | | 227.996 |
| fuan | 64123 | 354.4415 | 1.0845701 | 62.299999 | 512.34998 | | | 409.685 |
| fuqing | 63847.5 | 890.28326 | 1.40316 | 48.900002 | 365.09 | | | 250.41 |
| fuding | 59428 | 391.12057 | 1.03277 | 57.650002 | 506.948 | | | 370.67099 |
| xiuyu | 59337 | 1687.6838 | 2.45279 | 41.349998 | 246.174 | | | 268.81699 |
| luoyuan | 90197.5 | 241.36363 | 0.81550097 | 43.849998 | 565.789 | | | 501.19601 |
| xiangcheng | 84203.5 | 1811.1554 | 2.8871 | 90.5 | 1239.5 | | | 1161.96 |
| licheng | 67490.5 | 1846.1666 | 2.2977901 | 71 | 359.92001 | | | 232.668 |
| jiaocheng | 63949 | 315.35458 | 0.72776401 | 63.450001 | 872.83099 | | | 695.69202 |
| zhaoan | 33721 | 506.60742 | 0.97834802 | 42.049999 | 252.244 | | | 310.79199 |
| liancheng | 63282 | 133.22993 | 0.77950299 | 42.900002 | 630.02002 | | | 564.495 |
| lianjiang | 63807.5 | 563.23779 | 1.0470901 | 44.450001 | 347.897 | | | 228.373 |
| shaowu | 72681 | 107.95733 | 0.52332503 | 67.650002 | 705.297 | | | 857.85498 |
| changle | 83957.5 | 1082.8313 | 1.61246 | 48.799999 | 407.19901 | | | 285.16699 |
| changting | 44210 | 172.18457 | 0.67838699 | 47.299999 | 583.91101 | | | 572.16101 |
| changtai | 90071 | 230.38889 | 1.16539 | 52.849998 | 359.62799 | | | 363.75 |
| minhou | 65804.5 | 314.10797 | 0.81273401 | 53.849998 | 318.32199 | | | 208.317 |
| minqing | 60915 | 220.00682 | 1.03065 | 38.75 | 560.71399 | | | 607.56299 |
| xiapu | 41224 | 325.38736 | 0.797203 | 45.450001 | 445.561 | | | 374.03101 |
| shunchang | 50345.5 | 120.4798 | 0.579849 | 48.849998 | 442.64801 | | | 474.099 |
| mawei | 162847 | 616.72595 | 0.86476898 | 72.550003 | 350.71399 | | | 209.94299 |
| longwen | 94767 | 1143.254 | 1.12453 | 87.199997 | 525.87097 | | | 315.69 |
| longhai | 73093.5 | 660.11407 | 1.33812 | 55.049999 | | 301.918 | 297.12201 | |
